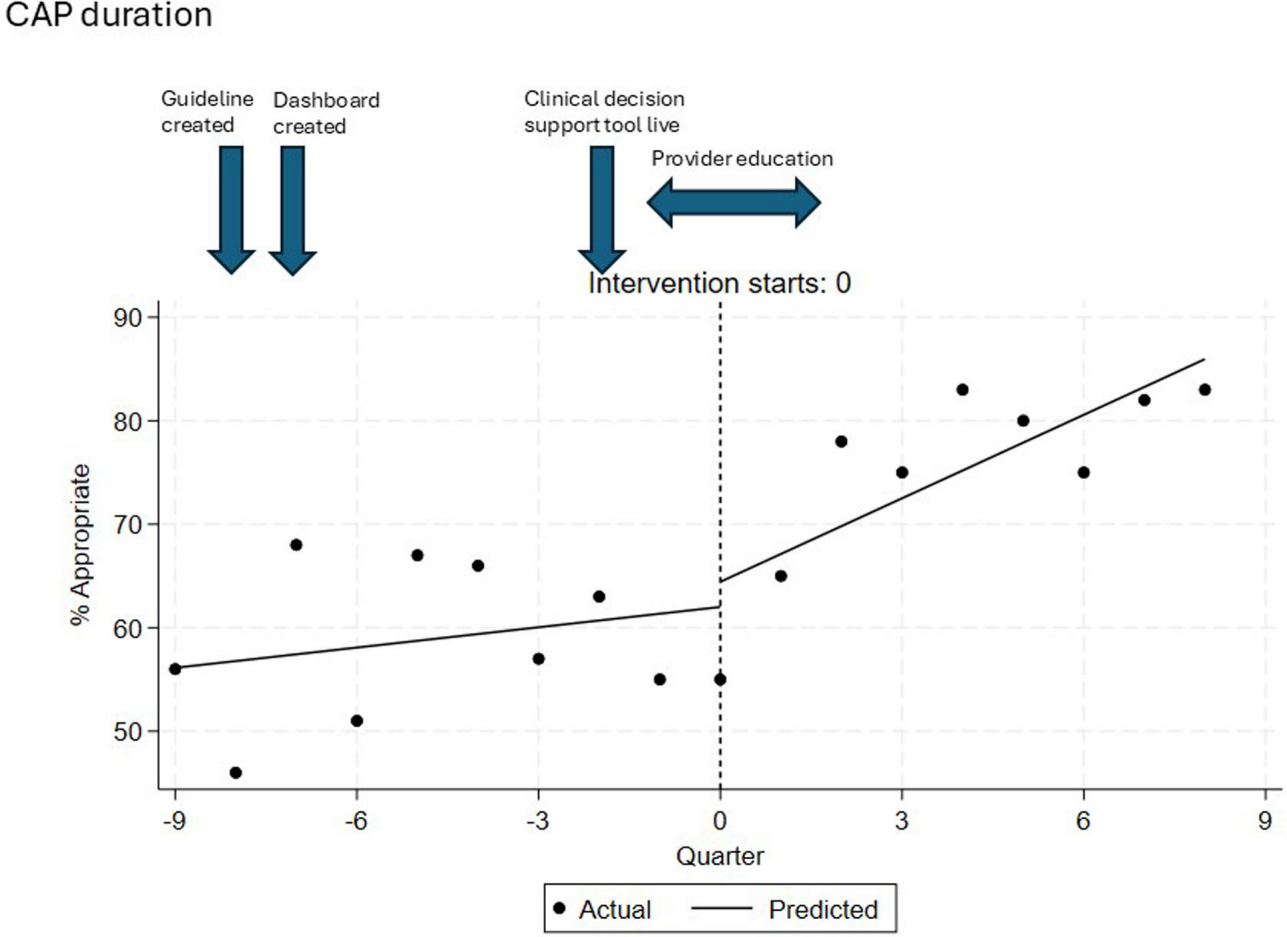# 76 Antibiotic Stewardship Capacity and Needs Among Consultant Pharmacists Serving Skilled Nursing Facilities in Philadelphia

**DOI:** 10.1017/ash.2026.10504

**Published:** 2026-06-23

**Authors:** Marten Hawkins, Nicholas Dillman, Lindsay Petty, Elizabeth Lloyd

**Affiliations:** 1 University of Michigan; 2 Michigan Medicine; 3 Pediatric Infectious Diseases

## Abstract

**Background:** Antimicrobial overuse increases antimicrobial resistance and adverse effects. Most antibiotics are prescribed in the ambulatory setting and up to half are inappropriate. We investigated the effect of a bundled stewardship intervention with active audit and feedback on antibiotic duration for two common infections, community acquired pneumonia (CAP) and skin and soft tissue infection (SSTI), in adult outpatients. **Methods:** From 2019-2021, institutional guidelines for management of CAP and SSTI were developed, and a dashboard to passively report data on provider-level antimicrobial durations for CAP and SSTI was created. From 2022-2023, clinical decision support tools promoting guideline-concordant antibiotic durations for both infection types were created in the Electronic Health Record. Education across all provider groups was completed throughout 2023. In the second quarter of 2023, personalized provider feedback reports (figure 1) on antimicrobial duration for CAP and SSTI were provided quarterly via electronic mail to individual providers. We performed an interrupted time series analysis of adult patients (age ≥21) prescribed an enteral antibiotic for CAP or SSTI within 3 days of a primary care encounter. The intervention period began one quarter after feedback was initially provided. Prescriptions of <3 days or <14 days were excluded as unlikely to represent uncomplicated infection. The primary outcome was proportion of patients prescribed appropriate antibiotic durations, defined as ≤5 days for CAP and ≤7 days for SSTI. **Results:** Eighteen quarters of data were analyzed (9 pre-intervention and 9 post-intervention). For SSTI (figure 2), there was no pre-intervention change in appropriate duration (p = 0.746). After intervention, there was an immediate increase in absolute proportion of appropriate antibiotic duration of 6.8 percentage points (p=0.009) with an ongoing increase of 1.25 percentage points each quarter (p=0.001). For CAP (figure 3), there was no pre-intervention change in appropriate duration (p = 0.44). There was no immediate change in absolute proportion of appropriate antibiotic duration (2.4%, p=0.698). After intervention, there was an ongoing increase in appropriate antibiotic duration of 2.69 percentage points each quarter (p=0.006), though not significantly different than the pre-intervention trend (p = 0.096). **Conclusion:** Clinician audit and feedback significantly improved antibiotic duration appropriateness for SSTI both immediately and progressively after implementation. Appropriate antibiotic duration for CAP also improved over time, though this was not significantly different than the pre-intervention trend. Active, individualized feedback to providers may be an effective strategy to improve appropriate antibiotic durations in ambulatory CAP and was an effective strategy for SSTI.

**Figure:**
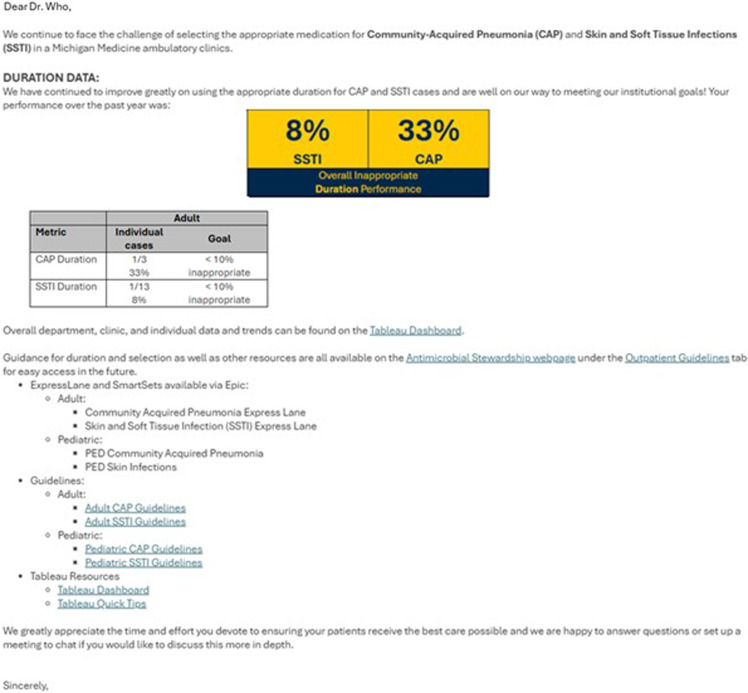

**Figure:**
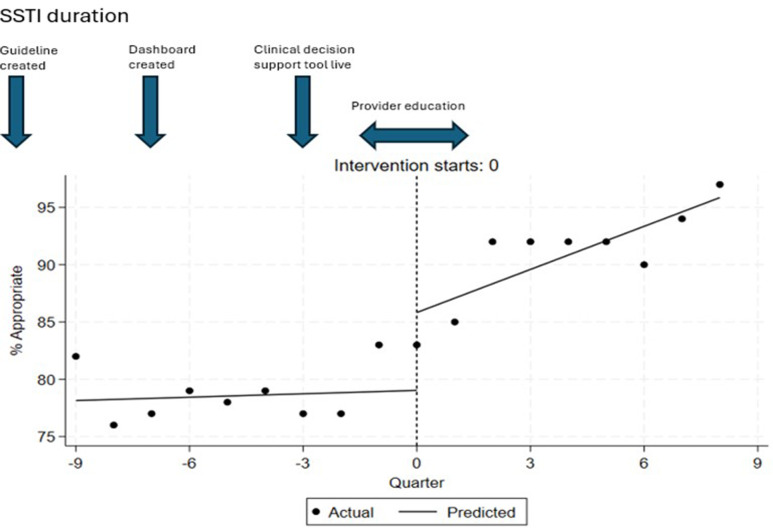

**Figure:**